# Prevention of sudden cardiac death in hypertrophic cardiomyopathy: Risk assessment using left atrial diameter predicted from left atrial volume

**DOI:** 10.1002/clc.23351

**Published:** 2020-03-07

**Authors:** Helen Mills, Kiri Espersen, Rebecca Jurlander, Kasper Iversen, Henning Bundgaard, Anna Axelsson Raja

**Affiliations:** ^1^ The Unit for Inherited Cardiac Diseases, Department of Cardiology Copenhagen University Hospital, Rigshospitalet, University of Copenhagen Copenhagen Denmark; ^2^ Department of Cardiology Copenhagen University Hospital, Herlev Hospital, University of Copenhagen Copenhagen Denmark

**Keywords:** echocardiography, implantable cardioverter‐defibrillator, risk model, risk prediction

## Abstract

**Background:**

Left atrial diameter (LAd) is included in the European Society for Cardiology's (ESC) risk model for assessment of sudden cardiac death (SCD) risk in hypertrophic cardiomyopathy (HCM), but the recommended measure of LA size is left atrial volume (LAv).

**Hypothesis:**

We hypothesized that LAv could be used instead of LAd in the HCM risk‐SCD model. We aimed to determine the relation between LAd and LAv and to assess the impact of using LAv instead of LAd.

**Methods:**

Echocardiographic measurements of anteroposterior LAd in the parasternal long‐axis window and LAv from Simpson's biplane method of disks were used. The 5‐year risk of SCD by *measured* LAd and by LAd *predicted* from LAv were estimated using the ESC risk‐SCD model.

**Results:**

In 205 HCM patients (age 56 ± 14 years, 62% male), the relation between LAd and LAv was linear. Median 5‐year risk of SCD was 2.4% (interquartile range [IQR]: 1.6; 3.8) using *measured* LAd and 2.4% (IQR: 1.6; 3.7) using *predicted* LAd. The correlation between the SCD risk assessed by *measured* vs *predicted* LAd was excellent (*r* ^2^ = 0.96). Use of *predicted* LAd resulted in four patients (2%) being recategorized between the moderate and high‐risk categories.

**Conclusions:**

The relation between LAd and LAv was linear with good agreement. On a population level, the correlation between the risk of SCD using *measured* LAd or LAd *predicted* from LAv was excellent. On a patient level, using LAd *predicted* from LAv resulted in the vast majority remaining in the same risk category; however, for a minority of patients, it changed the recommendation.

## 1 INTRODUCTION

Hypertrophic cardiomyopathy (HCM) is an autosomal dominantly inherited disease characterized by hypertrophy of the left ventricle not explained solely by loading conditions. HCM is most commonly caused by mutations in cardiac sarcomere genes that histologically lead to myocyte hypertrophy and disarray as well as interstitial fibrosis. The clinical manifestations of HCM are variable ranging from no symptoms to dyspnea, chest pain, syncope, and sudden cardiac death (SCD).^1^, ^2^ HCM is estimated to occur in at least 1 in every 500 adults and is the most common cause of SCD in young individuals, but SCD occurs in all age groups.^3^, ^4^

Assessing the risk of SCD plays a pivotal role in the management of HCM patients and regular assessments every 12 to 18 months are clinical routine to identify the high‐risk patients qualifying for an implantable cardioverter‐defibrillator (ICD). A clinical risk prediction model assessing the 5‐year risk of SCD was presented in 2014 as a result of the HCM risk‐SCD study.^5^ This model is used in daily clinical practice as an important tool in the risk assessment, and the risk estimate is of major importance for the decision of ICD implantation. The model has since been validated in other studies, most recently in the large multicenter EVIDENCE‐HCM study,^6^ and has been shown to predict SCD better than in previous models^7^, ^8^, ^9^ and is now integrated in the European Society of Cardiology (ESC) guidelines.^10^

The risk‐SCD model includes seven clinical predictors that have been shown to independently predict SCD. One of these predictors is left atrial diameter (LAd).^5^, ^11^ However, LAd is an one‐dimensional estimate of the three‐dimensional structure of the left atrium, and recent echocardiographic guidelines for chamber quantification by echocardiography suggest biplane estimation of left atrial volume (LAv) as the most accurate method to estimate left atrial size.^12^, ^13^, ^14^ Consequently, LAv is now the recommended measurement and the measurement that is included in most standard echocardiographic reports.

The first aim of this study was to determine the relation between LAd and LAv in HCM. Second, we aimed to assess if the use of LAd *predicted* from LAv using this relation instead of *measured* LAd affects the SCD risk estimate.

## 2 METHODS

### 2.1 Study design and patient population

The study is a retrospective, observational study. Patients were included if they had been diagnosed with HCM in the outpatient clinic at the Unit for Inherited Cardiac Diseases at Rigshospitalet, Copenhagen, Denmark (2011‐2017). The diagnosis was made according to standard criteria being maximal wall thickness of ≥ 15 mm (≥13 mm for first‐degree relatives) in one or more left ventricular myocardial segments that were not explained solely by loading conditions.^10^ Echocardiographic data were unavailable for 13 patients and they could thus not be included in the study. The initial cohort consisted of 230 patients. Exclusion of a total of 25 patients was based on the following: extreme values of maximal wall thickness (MWT) (n = 1), left ventricular outflow tract gradient (LVOT) (n = 1), or age (n = 6) according to recent ESC guidelines on diagnosis and management of HCM^10^ and due to one or more of the following findings; previous aborted sudden cardiac death (aSCD) (n = 7), appropriate ICD shock (defined as shock due to ventricular tachycardia (VT) or ventricular fibrillation (VF) (n = 8) as well as patients with sustained VT (sVT) with hemodynamic compromise (n = 7), since those are considered absolute indications for a secondary prophylactic ICD (Figure S1). In addition, we conducted a sensitivity analysis with calculations excluding patients with previous septal reduction therapy, due to the ESC guidelines' recommendations to use the risk model cautiously on those patients. The Danish Data Protecting Agency and the National Board of Health in Denmark approved this study.

### 2.2 Registration process and variables included in the model

Patient data were registered retrospectively in the REDCap database for rare and inherited heart diseases. The registration included demographics and lifestyle data, diagnostic data and results of former examinations, information on genetic status as well as data from the most recent outpatient visit including echocardiography, Holter monitoring, electrocardiography (ECG), clinical assessments, and information on family history. Variables included in the ESC HCM risk‐SCD model are age, history of unexplained syncope, family history of SCD in greater than first‐degree relative <40 years or sudden death in a first‐degree relative with the same diagnosis regardless of age, left ventricular MWT, LVOT, presence of nonsustained ventricular tachycardia (nsVT) on Holter monitoring, and left atrial anterior‐posterior diameter.^5^

### 2.3 Clinical work‐up for patients with HCM

The assessment in the outpatient clinic of a patient with suspected HCM or a relative included the clinical history including thorough evaluation of any previous cardiovascular symptoms, such as unexplained syncope, chest pain, shortness of breath and palpitations, compiling a pedigree, physical examination, 12‐lead ECG, and echocardiography. Results of a 48‐hour Holter monitoring were assessed and thoroughly examined for occurrence of nsVT and sVT as well as other abnormalities. Echocardiography was performed on a Vivid E9 (GE Healthcare, Norway) or a Philips iE33 (Philips Healthcare, Best, the Netherlands). Images were stored digitally for offline analysis using Echopac (GE Healthcare, Norway) or Xcelera (Philips Healthcare, Best, the Netherlands).

Antero‐posterior LAd was measured in the parasternal long‐axis window (PLAX) and LAv was estimated using Simpson's biplane method of disks in the apical two‐ and four‐chamber views. Both measurements were conducted at ventricular end‐systole on the frames just prior to mitral valve opening.^12^

### 2.4 Statistical methods and data presentation

Normally distributed data are presented as mean ± 1 SD and nonnormally distributed data as median and interquartile range (IQR). Categorical variables are reported as percentages. The relation between LAd and LAv was assessed by regression models for squared, cubic, linear, and logarithmic models. The *predicted* LAd was calculated from LAv with the generated model. The estimated 5‐year risk of SCD was calculated with the ESC HCM risk‐SCD model using both *measured* LAd and LAd *predicted* from LAv.^5^ Between‐group differences were analyzed with the unpaired Student's *t* test or χ^2^‐test, depending on the type of variable and distribution. A *P*‐value < .05 was considered significant. To evaluate interobserver variability, two independent observers conducted repeat measurements of LAd and biplane LAv for 20 randomly selected patients without knowledge of the results obtained by the other observer. Interobserver variability was determined by the intraclass correlation coefficient (ICC). The ICC coefficient for agreement was interpreted as poor (ICC < 0.5), moderate (0.5 ≤ ICC < 0.75), good (0.75 ≤ ICC < 0.9), and excellent (ICC ≥ 0.9). Statistical analyses were performed using IBM SPSS Statistics 22.

## 3 RESULTS

### 3.1 Baseline demographics and clinical characteristics

Clinical and demographic characteristics of the study population are summarized in Table 1. We included 205 adult HCM patients (age 56 ± 14 years, 62% male). Mean *measured* LAd was 42 ± 7 mm and mean LAv was 88 ± 33 mL. A comparison of baseline characteristics for patients included in and excluded from the study can be seen in Table S1. A history of unexplained syncope was less common in included patients (11 vs 28%). By design, malignant ventricular arrhythmia was more frequent in excluded patients, but otherwise in‐ and excluded patients were comparable.

**Table 1:** Baseline characteristics of 205 patients with HCM

Demographic characteristics	
Age at evaluation, (years)	56 ± 14
Gender	
Male: no. (%)	126 (62)
Medical history	
Disease causing mutation: no. (%)	82 (40)
Family history of SCD: no. (%)	39 (19)
Unexplained syncope: no. (%)	23 (11)
Previous septal reduction therapy	
Alcohol ablation: no. (%)	39 (19)
Myectomy: no. (%)	10 (5)
Echocardiographic and Holter monitoring data	
Nonsustained VT: no. (%)	71 (35)
LVOT Valsalva: mm Hg median(IQR)	17 (8;22)
Maximal wall thickness: mm	20 ± 6
LA diameter *measured*: mm	42 ± 7
LA volume: mL	88 ± 33

Abbreviations: LA, left atrial; LVOT, left ventricular outflow tract; SCD, sudden cardiac death; VT, ventricular tachycardia.

The sensitivity analysis (Table 2) showed that exclusion of patients with septal reduction therapy did not alter our results, and therefore we chose to retain those patients in our study population for further analysis. Furthermore, the risk model has recently been validated in patients treated with alcohol septal ablation.^8^

**Table 2:** Sensitivity analysis excluding patients with previous septal reduction therapy

	Including patients with precious septal reduction therapy n = 205	Excluding patients with previous septal reduction therapyn = 163
Model for *predicted* LAd	LAd (mm) = 28 + 0.16 × LAv (mL)	LAd (mm) = 27 + 0.16 × LAv (mL)
Number of recategorized patients, n (%)	10 (5%)	8 (5%)

Abbreviations: LAd, left atrial diameter; LAv, left atrial volume.

### 3.2 Relation between LAd and LAv

**Figure 1:**
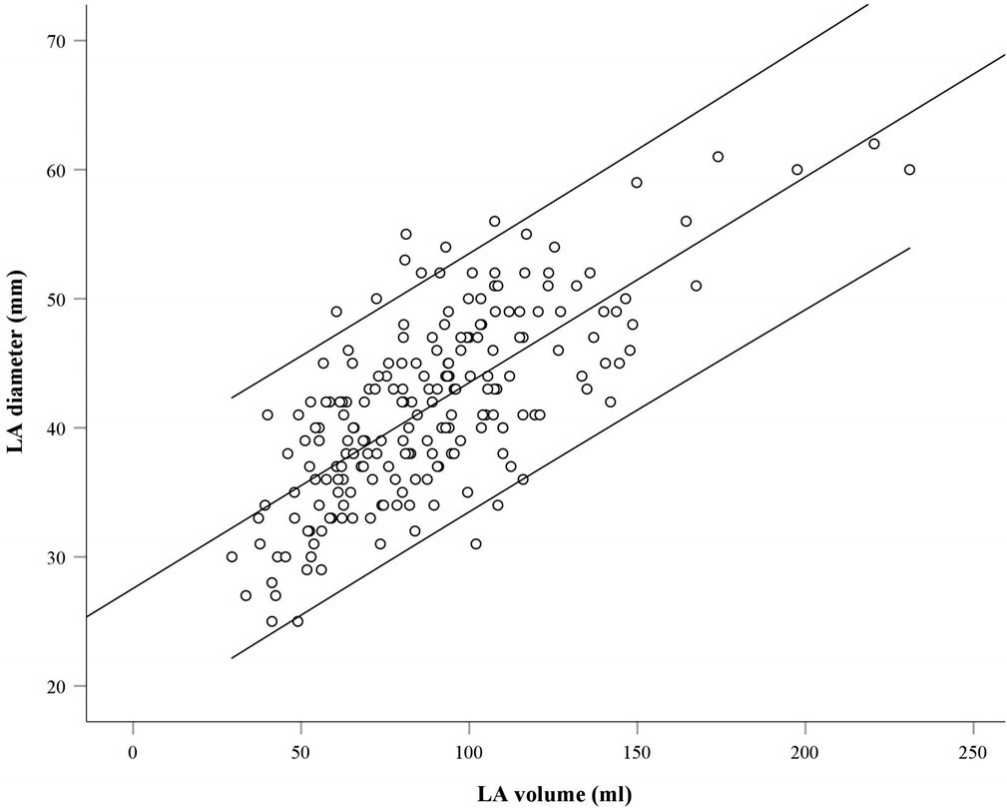
Relation between left atrial (LA) volume (mL) and LA diameter (mm) in a linear regression analysis with a linear regression line as well as 95% confidence limits

The assessment of the relation between LAd and LAv showed that the best fitting model was a linear regression model (Figure 1). This allowed us to generate a model for a *predicted* LAd from LAv (LAd [mm] = 28 + 0.16 × LAv [mL]).

When using the model for a *predicted* LAd from LAv the ESC HCM risk‐SCD model^5^ would appear as follows:

Prognostic Index = 0.15939858 × Maximal wall thickness (mm) − 0.00294271 × Maximal wall thickness^2^ (mm^2^) + 0.0259082 × (28 + 0.16 × LAv [mL]) + 0.00446131 × Maximal left ventricular outflow tract gradient (mm Hg) + 0.4583082 × Family history SCD + 0.82639195 × NSVT +0.71650361 × Unexplained syncope − 0.01799934 × Age at clinical evaluation (years).

Probability of SCD at 5 years = 1 − 0.998 × exp^(PrognosticIndex)^.

Mean *predicted* LAd in HCM patients was 42 ± 5 mm. Agreement between *measured* LAd and LAd *predicted* from LAv can be seen in Figure S2. The interobserver analysis regarding measurement of LAd and LAv showed excellent agreement with an ICC of 0.95 for LAd and 0.97 for LAv (Supplemental Table 3).

### 3.3 Impact of the use of LAd predicted from LAv instead of measured LAd on the assessment of SCD risk

**Figure 2:**
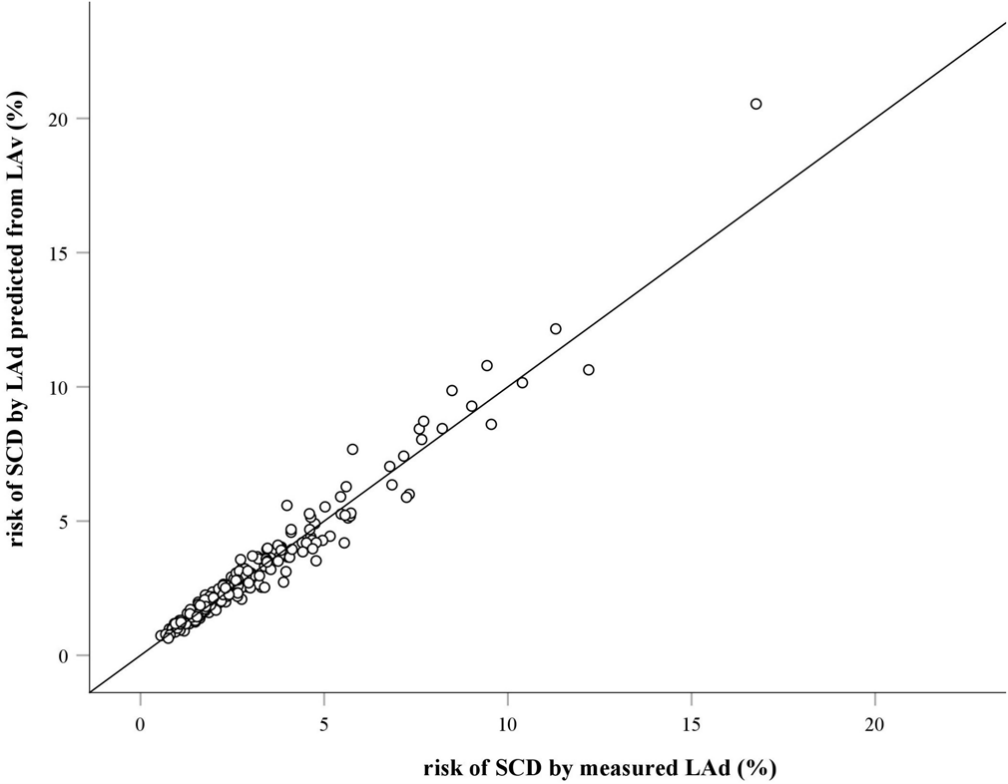
Agreement between the SCD risk scores (%) using *measured* LAd (mm) and LAd *predicted* from LAv (mm), respectively. The agreement is illustrated in a scatter plot with a 45° line of equality. LAd, left atrial diameter; LAv, left atrial volume; SCD, sudden cardiac death

**Figure 3:**
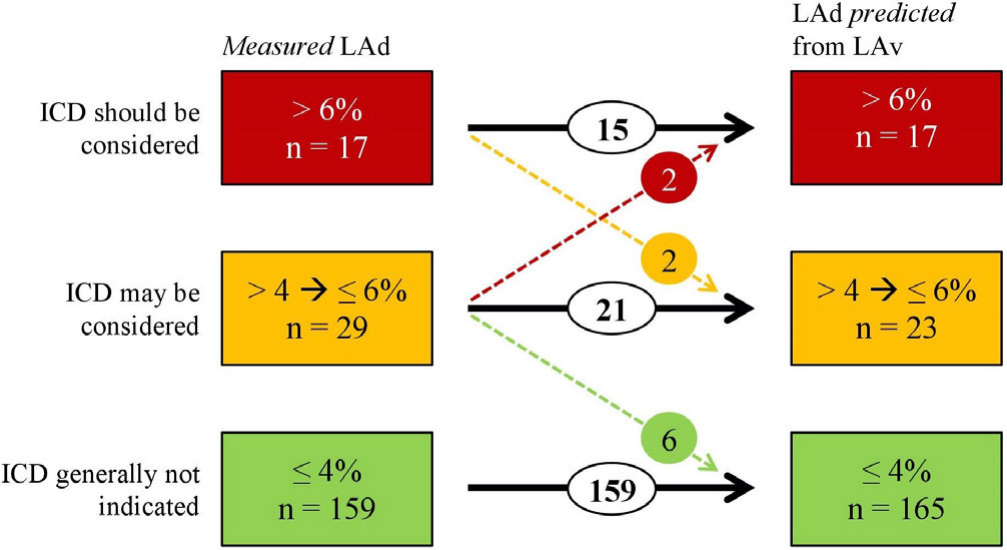
Recategorization according to ICD recommendation. Recategorization of patients into a different category of SCD risk (%) and ICD recommendation, when using *measured* LAd (mm) and LAd *predicted* from LAv (mm), respectively. The colors green, yellow, and red symbolize the lowest, intermediate, and high‐risk categories. LAd, left atrial diameter; LAv, left atrial volume; ICD, implantable cardioverter‐defibrillator

When applying our model and estimating the risk of SCD using *measured* LAd and LAd *predicted* from LAv respectively, the correlation between the two models was excellent (*r* ^2^ = 0.96, Pearson's correlation coefficient) (Figure 2). Median 5‐year risk of SCD was 2.4% (IQR: 1.6; 3.8) using *measured* LAd and 2.4% (IQR: 1.6; 3.7) using LAd *predicted* from LAv. When using *measured* LAd in the ESC HCM risk‐SCD model, 17 patients were considered at high risk, 29 patients were considered at moderate risk, and 159 patients at low risk (Figure 3). When applying the *predicted* LAd instead of *measured* LAD, a total of 10 patients (5%) were recategorized into a different category of SCD risk (Figure 3).

Four of the 10 patients were recategorized between moderate and high‐risk categories. Two patients were upgraded from “ICD may be considered” to “ICD should be considered” and two patients were recategorized from “ICD should be considered” to “ICD may be considered.” The detailed description of baseline characteristics and outcome of those four patients are given in Table S2.

The remaining six patients were recategorized between moderate and low‐risk categories. They had a median SCD risk of 4.1% (IQR: 4.0; 4.3) when using *measured* LAd and a median SCD risk of 3.8% (IQR: 3.7; 3.9) when using LAd *predicted* from LAv.

When reviewing the medical charts of the 10 recatergorized patients, we observed that none of them died during a mean follow‐up time of 3.7 years.

## 4 DISCUSSION

In this HCM cohort of more than 200 patients the major findings were that the relation between LAd and LAv was linear with a good agreement and that the correlation between the calculated risk scores using *measured* LAd vs LAd *predicted* from LAv was excellent on a population level and that only few patients (5%) were recategorized into a different category of SCD risk when using our model. Using LAd *predicted* from LAv would only have changed the clinical management regarding recommendation of ICD implantation in four patients.

The relationship between a dimension and a volume would typically not be expected to be a linear relationship. Our findings were however in accordance with a previous study conducted by Canciello et al. who found that LAv can be estimated from LAd using a linear model when using Simpson's biplane method of disks for LAv estimation in an unselected population with normal as well as abnormal echocardiographic findings and thus not specific for HCM patients.^15^ Their approach was the opposite of ours since they sought to estimate LAv from LAd, yet their finding of linearity supports our findings.

LAd as opposed to LAv was used in the ESC HCM risk‐SCD study since LAd was the measurement that was previously generally used and therefore was available for all patients.^5^ However, in the meantime the more accurate estimate of left atrial size, LAv, is primarily used and there is universal agreement that LAv is the most reliable measurement for echocardiographic assessment of the size of the left atrium.^12^ LAd measurements have a high reproducibility, but do not take into account that the left atrium may not enlarge equally in all dimensions.^16^

Our interobserver analysis showed good agreement for both LAd and LAv. Since biplane estimation of LAv is based on fewer geometrical assumptions, it is likely to be more accurate. Naturally, the clinical inaccuracy of the method used to measure LA size will be reflected to some degree in the risk estimate for SCD.

The most common causes of left atrial enlargement in HCM are systolic anterior movement (SAM) with related mitral regurgitation and elevated left ventricular filling pressures (diastolic dysfunction). Several prospective studies have shown that left atrial enlargement is present in approximately one‐third of patients with HCM and that left atrial size provides important prognostic information.^17^, ^18^, ^19^ Patients with HCM and left atrial enlargement are more likely to suffer from major adverse cardiac and cardiovascular events, including atrial fibrillation, stroke, sudden death, and congestive heart failure than patients without left atrial enlargement.^20^ Losi et al. imply in their study that the prognostic power of LAd is lower than the prognostic power of LAv, and that LAv may be a more reliable predictor of prognosis in HCM patients.^17^ The prognostic importance of left atrial size emphasizes the need for an accurate method for determination of the size. Three‐dimensional assessment of left atrial size using cardiac magnetic resonance (CMR) imaging or 3D echocardiography is not always as easily available and applicable as 2D echocardiography in a clinical setting.

We showed that using LAd *predicted* from LAv had a good agreement with the original risk prediction model using *measured* LAd, on a population level. On an individual level, only few patients were recategorized into a different category of SCD risk when using LAd *predicted* from LAv. To put the observed change in SCD risk score when using LAd *predicted* from LAv instead of LAd in perspective, we must also consider that the seven clinical predictors used in the SCD risk score are not constant over time for the individual patient. The literature on how much SCD risk scores vary from one outpatient visit to another is scarce, but clinical experience shows that the risk estimation for one patient may vary from one visit to another due to inaccuracy of, for example, MWT measurements and/or inaccuracy of measurement of the maximal LVOT gradient as well as physiological variability. When considering this variability, the recategorization of patients in our study, which is due to minor changes in the estimated SCD risk, may be of no clinical significance regarding the risk estimation of SCD.

Our study was not designed to assess change in the real, observed rate of SCD by including follow‐up data and is thus simply an assessment of the effect on the risk score for SCD. Larger studies, preferably prospective, are needed to confirm that LAv and LAd can be used interchangeably for the assessment of SCD risk.^21^

The two currently recommended models for risk stratification in the European and American guidelines, respectively only have moderate ability to discriminate patients with a high risk of SCD from patients with a low risk.^22^ Particularly, it is striking that most patients who experience an event (aSCD or SCD) stem from the low‐risk group, since the group of patients considered at a low risk is large (in our study 78%) in comparison with the moderate and high‐risk groups. Consequently, it is of great importance that we continuously work on improving the risk prediction models, using the most updated measurement methods for the included parameters and identify additional risk factors that may contribute in making risk prediction more accurate.

## 5 LIMITATIONS

The model derived for the relation between LAd and LAv in our population of 205 patients requires validation in a separate population of patients with HCM to be implemented in clinical practice. Furthermore, LAv has been shown to be less reproducible compared with LAd. In addition, in spite of LAv being the recommended measurement for estimation of left atrial size, a two‐dimensional measurement of LAv will still include geometrical assumptions of the left atrium being a cylindrical shape and it has been shown that two‐dimensionally estimated volume measurements consistently underestimate left atrial volume compared with three‐dimensional estimates.^23^

## 6 CONCLUSION

In patients with HCM, we found the relation between LAv and LAd to be linear with a good agreement. The correlation between the risk of SCD using either directly *measured* LAd or LAd *predicted* from LAv is excellent on a population level. On a patient level, using LAd *predicted* from LAv in the current ESC HCM risk‐SCD model resulted in the vast majority of patients, that is, the low‐risk group, remaining in the same category of SCD risk, however for a small subset (2%) of patients with a SCD risk score close to the 6% using LAd *predicted* from LAv may change the recommendation between the categories “ICD *may*” or “ICD *should* be considered.”

## CONFLICT OF INTEREST

The authors declare no potential conflict of interest.

## Supporting information


**Figure S1** Patient selection for the study population. Eight patients were excluded due to MWT, LVOT gradient, LAd or Age being out of range according to recent ESC guidelines. Furthermore, 17 patients were excluded due to previous aSCD, ICD shock or sVT. MWT Maximal wall thickness; LVOT Left ventricular outflow tract; LAd: Left atrial diameter; SCD Aborted sudden cardiac death; ICD Implantable cardioverter‐defibrillator; sVT Sustained ventricular tachycardia

**Figure S2** Agreement between *measured* LAd (mm) and LAd *predicted* from LAv (mm). Agreement between *measured* LAd (mm) and LAd *predicted* from LAv (mm) illustrated in a scatter plot with a 45° line of equality. LAd: Left atrial diameter; LAv: Left atrial volume

**Table S1** Comparison of baseline characteristics in included vs excluded patients
**Table S2** HCM risk‐SCD score; recategorization of four patients between moderate and high‐risk categories
**Table S3** Intraclass correlation coefficients for LAd and LAv measured by clinician 1 and clinician 2
