# Supplementary figures and images for: Prevention of sudden cardiac death in hypertrophic cardiomyopathy: Risk assessment using left atrial diameter predicted from left atrial volume
Source: Clin Cardiol. 2020 Mar 7;43(6):581–6. doi: 10.1002/clc.23351 (PMC7298985; doi:10.1002/clc.23351)

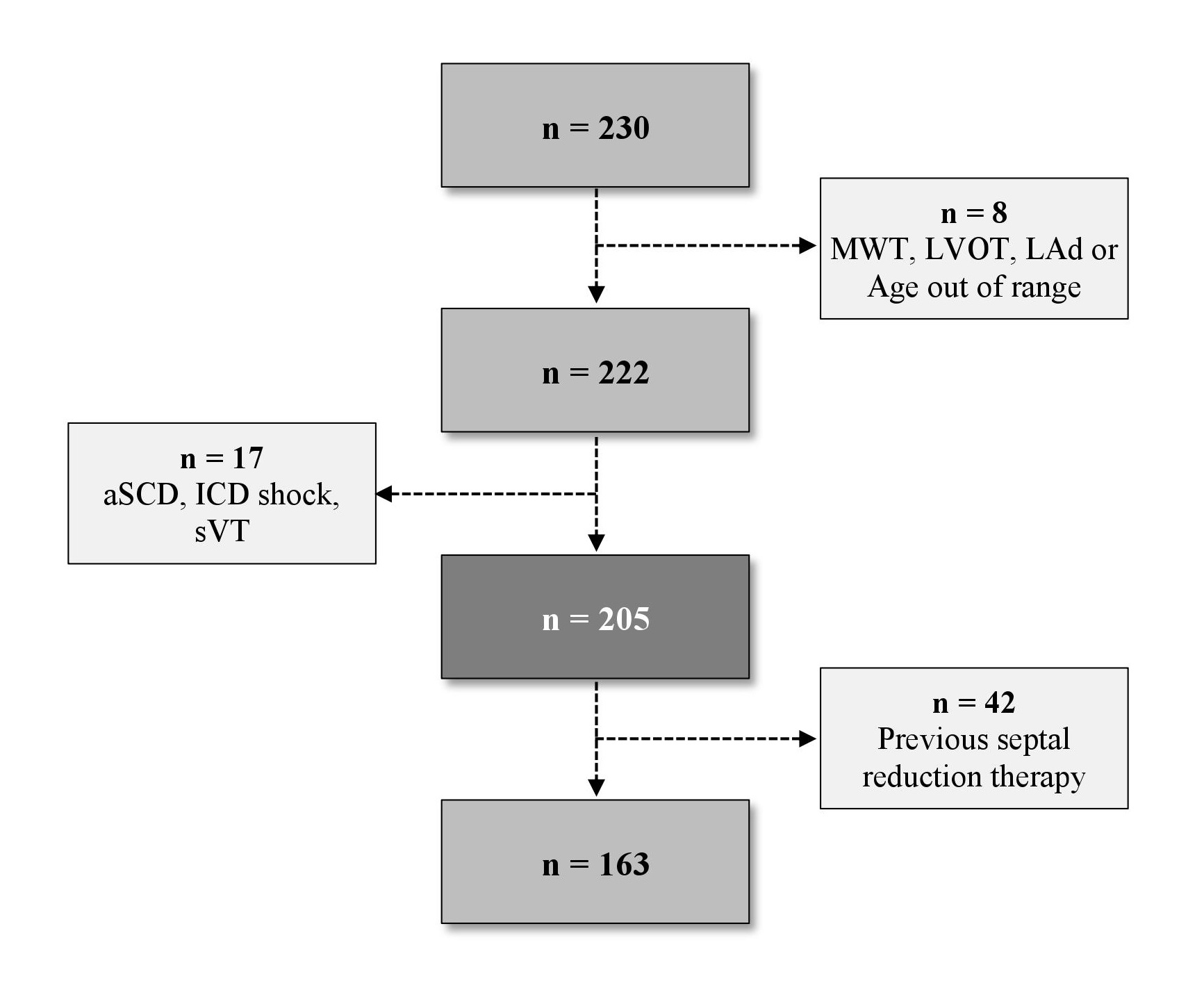

Supplement: Supplementary file 1 — Figure S1 Patient selection for the study population. Eight patients were excluded due to MWT, LVOT gradient, LAd or Age being out of range according to recent ESC guidelines. Furthermore, 17 patients were excluded due to previous aSCD, ICD shock or sVT. MWT Maximal wall thickness; LVOT Left ventricular outflow tract; LAd: Left atrial diameter; SCD Aborted sudden cardiac death; ICD Implantable cardioverter‐defibrillator; sVT Sustained ventricular tachycardia [file CLC-43-581-s001.jpg]

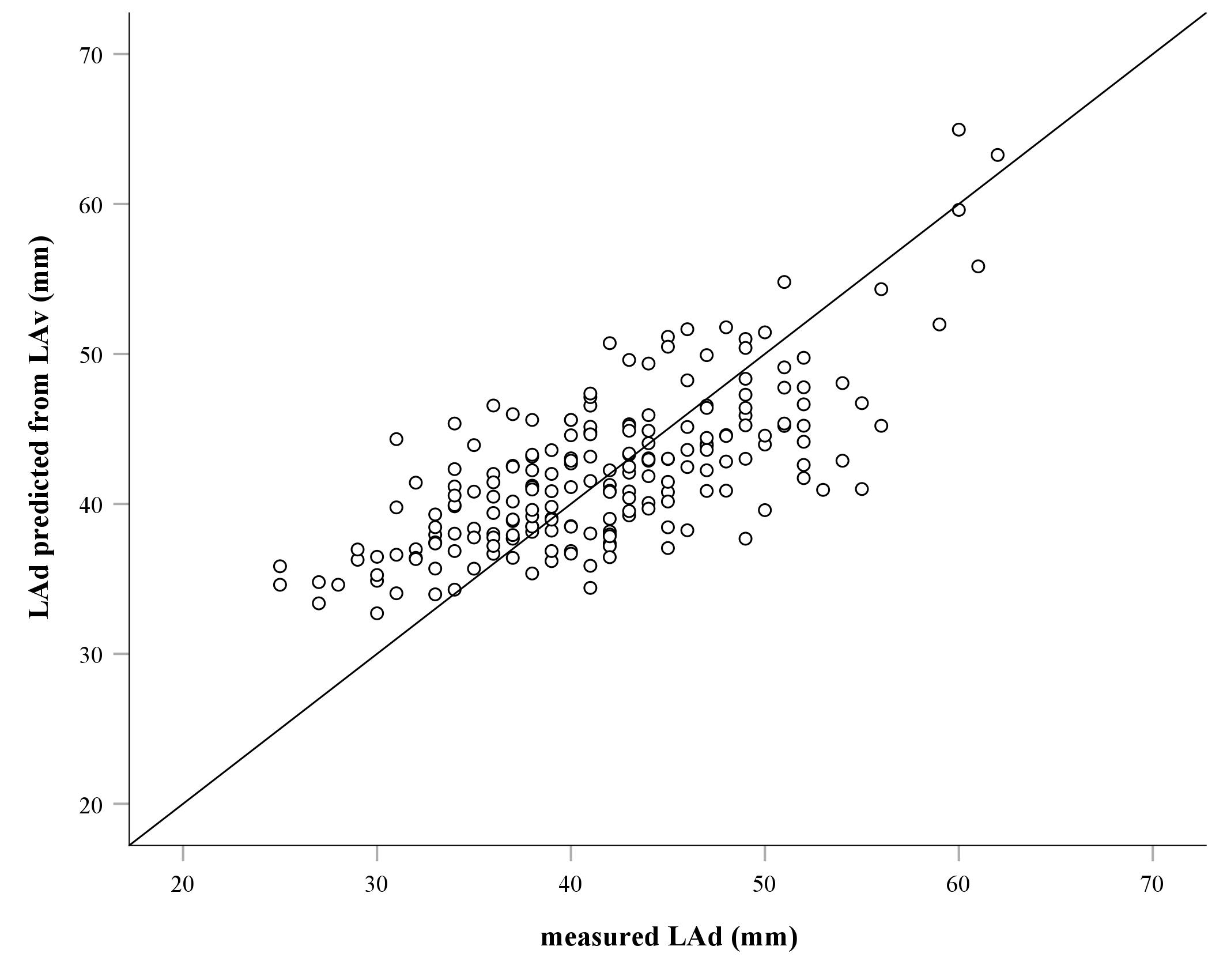

Supplement: Supplementary file 2 — Figure S2 Agreement between measured LAd (mm) and LAd predicted from LAv (mm). Agreement between measured LAd (mm) and LAd predicted from LAv (mm) illustrated in a scatter plot with a 45° line of equality. LAd: Left atrial diameter; LAv: Left atrial volume [file CLC-43-581-s002.jpg]
