# Supplementary material for: Prevention of sudden cardiac death in hypertrophic cardiomyopathy: Risk assessment using left atrial diameter predicted from left atrial volume
Source: Clin Cardiol. 2020 Mar 7;43(6):581–6. doi: 10.1002/clc.23351 (PMC7298985; doi:10.1002/clc.23351)
Supplement: Supplementary file 3 — Table S1 Comparison of baseline characteristics in included vs excluded patients Table S2 HCM risk‐SCD score; recategorization of four patients between moderate and high‐risk categories Table S3 Intraclass correlation coefficients for LAd and LAv measured by clinician 1 and clinician 2 [file CLC-43-581-s003.docx]

**Supplemental tables**

| **Supplemental table 1** Comparison of baseline characteristics in included vs excluded patients | | | |
| --- | --- | --- | --- |
|  | | | |
| **Demographic Characteristics** | **Included patients n = 205** | **Excluded patients n = 25** | **p-value** |
| Age at evaluation – years | 56±14 | 57±20 | 0.73 |
| Gender |  |  |  |
| Male – no. (%) | 126 (61) | 12 (52) | 0.36 |
| **Medical History** | | | |
| Disease causing mutation – no. (%) | 82 (40) | 7 (28) | 0.25 |
| Family history of SCD – no. (%) | 39 (19) | 2 (8) | 0.17 |
| Unexplained syncope – no. (%) | 23 (11) | 7 (28) | **0.02** |
| Previous septal reduction therapy |  |  |  |
| Alcohol ablation – no. (%) | 39 (19) | 8 (32) | 0.13 |
| Myectomy – no. (%) | 10 (5) | 2 (8) | 0.51 |
| Aborted SCD – no. (%) | 0 (0) | 7 (28) | **<0.0001** |
| Appropriate ICD therapy – no. (%) | 0 (0) | 7 (28) | **<0.0001** |
| Sustained VT – no. (%) | 0 (0) | 7 (28) | **<0.0001** |
| **Echochardiographic and Holter data** | | | |
| Non-sustained VT – no. (%) | 71 (35) | 11 (44) | 0.36 |
| LVOT Valsalva – mmHg *median(IQR)* | 17 (8;22) | 19 (11;22) | 0.16 |
| Maximal wall thickness – mm | 20±6 | 21±7 | 0.56 |
| LA diameter *measured* – mm | 42±7 | 42±8 | 0.71 |
| LA volume – ml | 88±33 | 91±31 | 0.95 |
| SCD: Sudden cardiac death; ICD: Implantable cardioverter-defibrillator; VT: Ventricular tachycardia; LVOT: Left ventricular outflow tract; LA: Left atrial | | | |

| **Supplemental table 2** HCM risk-SCD score; recategorisation of four patients between moderate and high-risk categories | | | | | | | | | | | | | | | | | | |
| --- | --- | --- | --- | --- | --- | --- | --- | --- | --- | --- | --- | --- | --- | --- | --- | --- | --- | --- |
|  |  | |  | |  | |  | |  | |  | |  | |  | |  | |
| **Age**  **(yr.)** | | **Gender**  **(m/f)** | | **MWT**  **(mm)** | | ***Measured***  **LAd**  **(mm)** | | **LAd *predicted* from LAv**  **(mm)** | | **SCD risk score with *measured* LAd**  **(%)** | | **SCD risk score with LAd *predicted* from LAv**  **(%)** | | **ICD implanted**  **(Yes/No)** | | **Shocks from ICD or other cardiac events***  **(Yes/No)** | | **Follow up time**  **(yr.)** |
| 55 | | m | | 20 | | 40 | | 45 | | 5.6 | | 6.3 | | No | | No | | 2 |
| 22 | | m | | 32 | | 34 | | 45 | | 5.8 | | 7.7 | | Yes | | No | | 5 |
| 49 | | f | | 26 | | 45 | | 37 | | 7.3 | | 6.0 | | Yes | | No | | 7 |
| 72 | | m | | 17 | | 55 | | 47 | | 7.2 | | 5.9 | | Yes | | No | | 14 |
| MWT: Maximal wall thickness; LAd: Left atrial diameter; LAv: Left atrial volume; SCD: Sudden cardiac death; ICD: Implantable cardioverter-defibrillator  *events during follow-up: Cardiac syncope, aborted SCD or SCD | | | | | | | | | | | | | | | | | | |

| **Supplemental table 3** Intraclass correlation coefficients for LAD and LAV measured by clinician 1 and clinician 2 | | | |
| --- | --- | --- | --- |
|  | | | |
|  | **LAD (mm)** | **LAV (ml)** |  |
| **ICC** | 0.95 | 0.97 |  |
| **95% ICC lower** | 0.85 | 0.92 |  |
| **95% ICC upper** | 0.98 | 0.99 |  |
| **95% ICC interval** | (0.85;0.98) | (0.92; 0.99) |  |
| LAd: Left atrial diameter; LAv: Left atrial volume; ICC: Intraclass correlation coefficient | | | |
